# Employment and occupation effects on late-life depressive symptoms among older Koreans: a cross-sectional population survey

**DOI:** 10.1186/s40557-016-0107-2

**Published:** 2016-05-14

**Authors:** Hyun Park, Young Hwangbo, Yong-Jin Lee, Eun-Chul Jang, Wook Han

**Affiliations:** Department of Occupational and Environmental Medicine, Soonchunhyang University Cheonan Hospital, 31, Soonchunhyang 6-gil, Dongnam-gu, Cheonan-si, Chungcheongnam-do 330-930 Republic of Korea; Department of Preventive Medicine, College of Medicine, Soonchunhyang University, 31, Soonchunhyang 6-gil, Dongnam-gu, Cheonan-si, Chungcheongnam-do 330-930 Republic of Korea

**Keywords:** Employment, Occupation, Late-life depression, Older Korean

## Abstract

**Background:**

The present study investigated the prevalence of depressive symptoms in older Koreans and identified associations between depressive symptoms and occupational factors.

**Methods:**

Data from the Korean National Health and Nutrition Examination Survey V (2010–2012) were used to analyze 7320 participants aged 55 years or older. Complex sample logistic regression analysis was performed after adjusting general characteristics to determine associations between depressive symptoms and occupational factors.

**Results:**

Among older Korean men, the prevalence of depressive symptoms in the employed and the non-employed groups were 9.9 % and 13.7 %, respectively. Employment status was significantly associated with depressive symptoms after adjusting for general factors (OR: 0.69, 95 % CI: 0.49–0.97). Among older Korean women, the prevalence of depressive symptoms in the employed and the non-employed groups were 17.4 % and 20.3 %, respectively, but employment status was not significantly associated with depressive symptoms. Second skill level occupational groups (clerks, plant and machine operators) in particular showed significantly lower prevalence of depressive symptoms than the non-employed group of men (9.3 % vs 13.7 %). By occupation type, the odds ratios were 0.31 (95 % CI: 0.10–0.97, clerks) and 0.47 (95 % CI: 0.23–0.86, plant and machine operators) adjusting for general factors.

**Conclusions:**

The employed group showed lower late-life depressive symptom prevalence than the non-employed group among older Korean men. In addition some second skill level occupations (clerks, plant and machine operators) were significantly associated with a lower risk of depressive symptoms after adjusting for general factors in older Korean men.

## Background

According to a report by the World Health Organization, depression is one of the most threatening disorders in the world. Based on incidence trends, it is predicted to become humanity’s most serious health issue by 2030 [1]. Moreover, late-life depression has now emerged as a serious disorder with the increase of older populations and extended life expectancies [2].

Late-life depressive symptoms represent a serious health issue negatively related to the active aging that is globally pursued. They have been reported to be closely associated with late-life quality [3–6]. The prevalence of late-life depressive symptoms in Korea is more than 2–4 % higher than other Asian countries [7]. Prevalence of late-life depressive disorders in Korea, including major and minor depressive disorders, was ≥ 10 %, which is higher than most Western countries. The study also suggested the possibility that the prevalence of minor depressive disorders may increase significantly in the future [8].

The working-age population is associated with the demographic structural change trend. Since Korea is currently experiencing the fastest population aging among all Organization for Economic Co-operation and Development (OECD) countries, its working-age population is also undergoing rapid aging [9]. According to the 2011–2020 Medium to Long-term Labor Supply-demand Forecast published by the Korean Employment Information Service, a division of the Korean Ministry of Employment and Labor, the working youth population (15–29 years old) is forecasted to decrease by 1.1 % per annum and the primary working-age population (30–54 years old) will also see a decrease of  1.29 million workers by 2020. Whereas the working older population (≥55 years old) is forecasted to show a sudden increase from 2011 to 2020 due to the entry of first generation baby-boomers into this age group, the increasing effect is expected to be amplified by life expectancy extension in the existing older population. In particular, elderly workers 55 years or older are expected to show a significant increase in numbers from 4.76 million in 2010 to 7.73 million in 2020 [10].

Depression affects not only workers’ health, but their labor productivity as well. According to Stewart et al., workers with depression have more loss productive time (mean 5.6 h/week) than those without depression (mean 1.5 h/week). Depression is associated with at-work performance deficits and productivity loss during work [11].

Conventional studies of older Korean workers have exclusively targeted analysis of policies and industrial accidents status; few studies targeted mental health effects involving employment status and occupations in older Koreans. Studies from abroad on mental health related to older person’s working status [12–14] and socially productive activities [15–17] have been relatively active. Therefore, considering the increasing trend of older workers in Korea and the significance of late-life depressive symptoms, an identification of the association between depressive symptoms and occupation-related factors in older Koreans is meaningful. The present study investigated the prevalence of depressive symptoms in Koreans who are 55 years or older and associations between depressive symptoms and employmental and occupational factors.

## Methods

### Study subjects

The Korean National Health and Nutrition Examination Survey V 2010–2012 (KNHANES V) is a nationwide health and nutrition survey representative of Korea using a rolling survey sampling wherein each year’s samples are completed by interview or self-report format, depending on the question. A stratified multistage-clustered design was used to collect a representative sample of the Korean population. Sample units were subjected to two-stage stratification. The cities and provinces of Korea were stratified. The general housing area was then stratified in to 26 levels by sex, age group and population ratio. Apartment complex areas were stratified in to 24 levels by price per square meter and average number of square meters per apartment. Thus 192 sample comprising 3840 households were randomly sampled every year. In total 25,534 people from 11,520 sample units were extracted in KNHANES V survey. Among the KNHANES V survey population of 25,534 people, this study involved 7320 participants who were over 55 years old and answered the questionnaire. The Institutional Review Board of Korea Centers for Disease Control and Prevention approved the KNHANES protocol and informed consent was obtained for all participants.

### Depressive symptoms

To clinically define the depressive symptoms, the following mental health question within the health survey portion of the KNHANES was used.

Question: In the past year, have you felt sadness or despair that continued for two weeks or longer which interfered with activities of daily living? Respondents answering “yes” were considered to have depressive symptoms.

### General characteristics

The general characteristics collected in the KNHANES V included the following. Age was categorized as 55–64 or ≥ 65. Educational level (primary school, middle school, high school, college) and household income (1Q [High], 2Q, 3Q, 4Q [Low]) were categorized into quartile. General health status was categorized as excellent/good or fair/bad. Exercise capacity and daily life capacity were categorized as yes (no problem or a little problem) or no (cannot) respectively. Sleep duration was categorized as ≤5 h, 6–8 h or ≥9 h per day. The volume and frequency of alcohol consumption were asked using closed-ended questions; therefore, to calculate weekly alcohol consumption, the mean volume and frequency of alcohol consumption were multiplied. The products were categorized as < 2.5 times or ≥2.5 times standard-sized drinks per week. Participants were categorized as current smoking, ex-smoking or non-smoking. We conducted all analyses separately for men and women.

### Occupational characteristics

Older workers’ employment status was classified into the employed or non-employed group, depending on whether they had done paid work for more than one hour during the past week at the survey time. Then, the employed group was further classified by occupation skill level and occupation type by referring to the International Labor Organization’s International Standard Classification of Occupations (ISCO) [18]. In the present study, occupational skill levels were divided into four categories (third and fourth skills, second skill, first skill and non-employed) by occupational competency. Then, occupation types were divided by skill level, using major occupational categories. According to ISCO the third and fourth skill occupation group included professionals and managers; second skill included clerks, service and sales workers, agricultural and fishery workers and plant and machine operators; and first skill included manual workers (Table 1). Moreover, working hours were divided into four groups of < 15, 15–29, 30–51 and ≥ 52 using the Labor Standards Act of Korea’s definition of part time as 15 h, OECD’s short-term working hour standard of 30 h [19], and the Labor Standards Act of Korea’s 52 h stipulated per week.

**Table 1:** Distribution of Occupation skill level and Occupation type by sex in older Koreans

Occupation skill level	Occupation type	Male		Female	
		*N*	(%)	*N*	(%)
Third/Fourth kill level	Professional, manager	230	7.0	51	1.3
Second skill level	Clerk	90	2.6	35	0.8
	Service and sales	188	7.9	335	9.7
	Agricultural and fishery	620	18.5	493	10.9
	Plant and Machine operator	385	15.7	59	1.7
First skill level	Manual worker	361	11.3	524	14.0
Unemployed		1304	37.1	2645	61.7
Total		3178	100	4142	100

### Statistical analysis

Because the KNHANES V uses a complex sample design, a survey module for complex samples and survey weights were applied in the present study. We conducted a descriptive analysis on the general and occupational characteristics of the participants surveyed. In order to analyze the characteristics of the study population as well as factors influencing depressive symptoms, complex samples *χ*^2^ and *t*-tests were performed to calculate estimated percentages. In addition, complex sample logistic regression was performed to examine adjusted odds ratios (OR) and 95 % confidence intervals (CI) of the depressive symptoms after correcting for general and occupational characteristics. All statistical analyses were performed using SPSS Version 19.0 (SPSS Inc., Chicago, IL, USA) and the level of significance was set at *p* <0.05.

## Results

### Associations between general characteristics and depressive symptoms in older Koreans

Among 7320 participants included in the analysis, there were 3178 men (43.4 %) and 4142 women (56.6 %) and their mean age was 67.2 years old. Household income, family members, general health status, exercise capacity, daily life capacity and sleep duration were statistically significant characteristics in both men and women but education status was significant only in men, whereas age, drinking and smoking were not significantly different. Men tended to have higher depressive symptom prevalence with less education status, whereas women’s depressive symptom prevalence was not significantly different. For household income, the depressive symptom prevalence was highest in 4Q (14.1 %, 23.0 %) in both men and women. This was followed by 1Q (11.8 %), 3Q (10.2) and 2Q (8.5 %) in men compared to 2Q (18.5 %), 3Q (16.4 %), and 1Q (15.1 %) in women. Depressive symptom prevalence was higher for older persons living alone than living together in men (19.2 % vs 10.8 %) and women (23.0 % vs 18.5 %). Fair and bad health status had higher depressive symptom prevalence than excellent and good health status in men (13.7 % vs 6.3 %) and women (21.4 % vs 12.0 %). Those who could exercise (men: 11.1 %, women: 18.7 %) and have an active daily life (men: 10.9 %, women: 18.4 %) had lower depressive symptom prevalence than those who could not. Concerning sleep duration, the depressive symptom prevalence of men and women was lowest in 6–8 h per week (9.8 %, 17.0 %) together. This was followed by ≥ 9 h (14.4 %, 20.2 %) and ≤ 5 h (15.8 %, 23.7 %) per week in men and women, respectively (Table 2).

**Table 2:** Associations between general characteristics and prevalence of depressive symptoms by sex in older Koreans

Total *N* = 7,320	Prevalence of depressive symptoms					
	Male			Female		
	Total	%	*p*-value^a^	Total	%	*p*-value^a^
	3178			4142		
Age (years)						
55–64	1376	11.6	0.582	1771	18.6	0.450
≥ 65	1802	10.9		2371	19.8	
Educational status						
≤ Primary School	1175	13.8	0.009	2850	19.9	0.507
Middle school	593	11.2		560	17.4	
High school	885	10.1		561	18.4	
≥ College	525	7.0		171	15.9	
Household income						
1Q (High)	630	11.8	0.024	660	15.1	< 0.001
2Q	670	8.5		717	18.5	
3Q	872	10.2		1070	16.4	
4Q (Low)	1006	14.1		1695	23.0	
Family members						
1 person	187	19.2	0.003	771	23.0	0.018
≥ 2 persons	2991	10.8		3371	18.5	
General health status						
Excellent/Good	1063	6.3	< 0.001	959	12.0	< 0.001
Fair/Bad	2115	13.7		3183	21.4	
Exercise Capacity						
Yes (Can)	3148	111.1	0.025	4055	18.7	< 0.001
No (Cannot)	30	25.6		87	45.6	
Daily life Capacity						
Yes (Can)	3126	10.9	< 0.001	4002	18.4	< 0.001
No (Cannot)	52	34.3		140	40.5	
Sleep duration						
≤ 5 h	573	15.8	0.003	1188	23.7	< 0.001
6–8 h	2314	9.8		2647	17.0	
≥ 9 h	291	14.4		307	20.2	
Drinking						
< 2.5 Times	1987	11.5	0.763	3949	19.3	0.698
≥ 2.5 Times	1191	11.0		193	17.9	
Smoking						
Current smoking	911	12.9	0.181	136	29.2	0.230
Ex smoking	1784	10.1		153	22.7	
Non-smoking	483	12.0		3853	18.7	

^**a**^ *χ* ^2^ test

### Associations between employment and occupational characteristics and depressive symptoms in older Koreans

Among the men, depressive symptom prevalence was significantly lower in the employed group (9.9 %) than the unemployed group (13.7 %). Depressive symptom prevalence was the lowest in the second skill level group (9.3 %), followed by first skill level (10.7 %), third and fourth skill level (10.9 %), and unemployed group (13.7 %). Among the women, depressive symptom prevalence was lower in the employed group (17.4 %) than the unemployed group (20.3 %) but it was not statistically significant. Depressive symptom prevalence was the lowest in the third and fourth skill level group (7.7 %), followed by second skill level (16.3 %), first skill level (20.1 %), and the unemployed group (20.3 %). Both men and women had the lowest depressive symptom prevalence in the 30–51 working hours (9.8 %, 17.1 % respectively), but it was not significant (Table 3).

**Table 3:** Associations between employment and occupational characteristics and prevalence of depressive symptoms by sex in older Koreans

Total *N* = 7,320	Prevalence of depressive symptoms					
	Male			Female		
	Total	%	*p*-value^a^	Total	%	*p*-value^a^
	3178			4142		
Employment status						
Employed	1874	9.9	0.012	1497	17.4	0.057
Unemployed	1304	13.7		2645	20.3	
Occupation skill level						
Third/Fourth skill level	230	10.9	0.068	51	7.7	0.030
Second skill level	1283	9.3		922	16.3	
First skill level	361	10.7		524	20.1	
Unemployed	1304	13.7		2645	20.3	
Working hours (hrs/wk)						
< 15	1351	12.2	0.298	2760	19.9	0.548
15–29	311	13.9		346	18.0	
30–51	943	9.8		699	17.1	
≥ 52	641	10.4		419	19.8	

^a^ *χ* ^2^ test

### Odds ratios of depressive symptoms associated with employment status in older Koreans

We identified how the risk of depressive symptoms differs in men and women by occupational factors. In older men, the risk of depressive symptoms in the employed group was significantly lower than the non-employed group (OR: 0.69, 95 % CI: 0.49–0.97) adjusting for general covariates (age, education status, household income, family members, general health status, exercise capacity, daily life capacity, sleep duration, drinking and smoking). In older women, the risk of depressive symptoms between the employed and the non-employed group was not significantly different after adjusting for general covariates (Table 4).

**Table 4:** Odds ratios of depressive symptoms associated with general characteristics and employment by sex in older Koreans

	Male		Female	
	OR	95 % CI^a^	OR	95 % CI^a^
Age (years)				
55–64	1.44	1.02–2.04	1.25	0.97–1.62
≥ 65	1.0		1.0	
Educational status				
≤ Primary School	2.12	1.26–3.54	0.92	0.53–1.59
Middle school	1.79	1.04–3.06	0.89	0.47–1.67
High school	1.57	0.95–2.60	1.01	0.55–1.82
≥ College	1.0		1.0	
Household income				
1Q (High)	0.82	0.52–1.30	1.50	1.04–2.15
2Q	0.68	0.43–1.06	1.04	0.72–1.50
3Q	0.58	0.37–0.93	1.22	0.84–1.76
4Q (Low)	1.0		1.0	
Family members				
1 person	1.74	1.09–2.78	1.11	0.87–1.43
≥ 2 persons	1.0		1.0	
General health status				
Excellent/Good	0.49	0.34–0.70	0.56	0.42–0.73
Fair/Bad	1.0		1.0	
Exercise Capacity				
Yes (Can)	1.02	0.32–3.26	0.46	0.27–0.77
No (Cannot)	1.0		1.0	
Daily life Capacity				
Yes (Can)	0.39	0.16–0.95	0.48	0.30–0.76
No (Cannot)	1.0		1.0	
Sleep duration				
≤ 5 h	1.45	1.02–2.08	1.44	1.18–1.76
6–8 h	1.0		1.0	
≥ 9 h	1.25	0.78–2.00	1.05	0.73–1.51
Drinking				
< 2.5 Times	1.03	0.75–1.42	1.08	0.66–1.77
≥ 2.5 Times	1.0		1.0	
Smoking				
Current smoking	0.91	0.58–1.42	1.48	0.89–2.45
Ex smoking	0.72	0.48–1.10	1.09	0.67–1.77
Non-smoking	1.0		1.0	
Employment status				
Employed	0.69	0.49–0.97	0.91	0.74–1.14
Unemployed	1.0		1.0	

^a^ 95 % confidence interval

### Odds ratios of depressive symptoms associated with occupational skill level and occupation type in older Korean men

In Model 1 of Table 5, general characteristics and working hours were adjusted to investigate the association between depressive symptoms and occupational skill levels in older Korean men. The results in Model 1 showed that all occupational skill levels had lower risks of depressive symptoms than the non-employed group. In particular, the second skill level showed the lowest risk compared to the non-employed group and it was statistically significant (OR: 0.48, 95 % CI: 0.27–0.86).

**Table 5:** Odds ratios of depressive symptoms associated with occupational factors in older Korean men

	Model 1^a^		Model 2^a^	
	OR	95 % CI^b^	OR	95 % CI^b^
Occupation skill level				
Third/Fourth skill level	0.88	0.35–2.18		
Second skill level	0.52	0.30–0.90		
First skill level	0.60	0.32–1.15		
Unemployed	1.000			
Occupation type				
Professional, manager			0.81	0.34–1.91
Clerk			0.30	0.09–0.95
Service and sales			0.50	0.22–1.12
Agricultural and fishery			0.55	0.30–1.02
Plant and Machine operator			0.45	0.23–0.85
Manual worker			0.57	0.29–1.10
Unemployed			1.000	

^a^Multivariate logistic regression analysis, adjusted for general characteristics + working time (hrs/wk)

^b^95 % confidence interval

In Model 2 of Table 5, general characteristics and working hours were adjusted to investigate the association between depressive symptoms and occupational type in older Korean men. The results in Model 2 showed that lower risks of depressive symptoms were seen in all occupations than the non-employed group. In particular, 2 s skill level occupations (clerks, plant and machine operators) showed significantly lower risks of depressive symptoms than the non-employed group. The odds ratios were 0.31 (95 % CI: 0.10–0.97, clerks) and 0.47 (95 % CI: 0.25–0.88, plant and machine operators) respectively.

## Discussion

The present study identified the association between depressive symptoms and employment by gender in older Korean people. Then, we further investigated what relationships exist with respect to occupational skill levels and occupational types. Older Koreans showed lower prevalence of depressive symptoms in the employed group compared with non-employed group. However, there was a significant difference between the two groups in men but not in women. In addition, when general covariates were adjusted, statistically significant results were seen only in men. This demonstrated that employment was associated with depressive symptoms in older Korean men. Subsequent analysis of occupational skill levels and occupational types in older men resulted in the risk of depressive symptoms being lower in all occupational skill levels and occupational types than the non-employed group, with second skill level occupations showing statistically significant results. These findings support the results of previous studies that older people with social participation showed relatively low CES-D scores [20]. In an older group facing critical life turning points, such as retirement or health deterioration, productive activities positively assisted mental health [21]. Calvo used data from an American Health and Retirement Study to report that paid work, exercise, marriage, education and household income in older people favorably influenced health [13]. In a recent study on persons 60 years or older, employment had a positive influence on physical and cognitive functions [22].

According to Sugihara et al., there were gender differences in the relationship between productive roles and depressive symptoms. Because social norms of productivity generally tend to be more emphasized in men than women, paid working is associated with reduced depressive symptoms in Japanese men but not women [23]. Another study found that social norms related to work roles prompted a gender difference in the psychological reaction to job loss. It may be attributed to differences in perception of occupational views [24]. Our study is consistent with results indicating gender-based differences in mental health benefits derived from employment.

Several studies found that employment status is an important factor for depression and mental health among older people [25–28]. There may be differences in the associations between employment status and mental health by occupation types because occupations have different levels of integration, job stress and income and some require specialized skill or knowledge. Thus, 2 s skill level occupations (clerks, plant and machine operators) provided mental benefit compared with the non-employed group. However, we did not observe mental benefit in the first, third and fourth skill level occupations.

One paper studied not only employment, but associations by occupational types of older workers as well. In a study that investigated the effects of employment and occupation on depression in elderly 65 years or older, Christ et al. reported that relative differences in depression were seen in certain occupations, with office workers (white-collar) and agricultural workers showing relatively lower levels of depression than workers with other occupations [14]. This was partially consistent with the results of our study that indicated clerks and plant and machine operators showed lower risk of depressive symptoms.

In the present study, the 30–51 h/week work group showed the lowest prevalence of depressive symptoms compared to part time (< 15, 15–29) and overtime (≥ 52). Virtanen et al. reported that middle-to-late aged workers (44–66 years old) who worked over 55 h per week showed increased depressive and anxiety symptoms than those who worked for 35–40 h per week [29]. Additionally, long working hours are also known to have a negative effect on suicidal ideation [30], depression [31] and mental health.

The clinical depressive symptoms that fall under the category of depressive disorders, but which fail to satisfy all diagnostic criteria of major depression can be classified as either minor depression or subsyndromal depression. Such symptoms, in comparison to not feeling depressed, can also diminish psychosocial functioning and cause negative outcomes and prognosis. According to Lyness et al., in a 1-year prognosis survey, those with minor and subsyndromal depression showed 12.6 and 6.1 fold higher morbidity rate respectively for major depression than the non-depressed group [32]. According to a study by Cuijpers et al., depressive symptoms represent the biggest risk factor for occurrence of depression. When depressive symptoms are present, the incidence rates at which those symptoms can progress to minor or major depression increase significantly [33]. This was consistent with preceding studies that indicated each disorder classified within depression represented part of a continuum [34, 35]. Another study found that depressive symptoms are key symptoms in determining depression compared to a group without depressive symptoms, those with depressive symptoms had twice as much possibility of developing major depression after two years [33]. Furthermore, when older persons suffer depression, there is a higher probability of being accompanied by chronic diseases. Depression alone can greatly diminish the life quality related to health [36, 37].

Late-life depression shows the highest prevalence rate among all late-life mental diseases. Over 50 % of late-life depression patients have symptoms such as lower cognitive function, somatization and anxiety symptoms, which show qualitatively different patterns of depression than appears in young to mid adulthood [38, 39]. According to a comparative study by Michell and Subramaniam, late-life depression after 60 years of age compared to middle age showed no difference in symptom alleviation by initial treatment, but high probability of recurrence [40]. In addition, late-life depression shows generally poor prognosis that includes a high suicide rate [38, 39]. Thus, several policies are needed to solve the problem of late-life depression and employment of older people because productive role in active aging is the preferred answer.

The limitations in the present study are as follows. First, the present study was a cross-sectional study therefore we could not determine a temporal causal relationship. Second, since the survey on depressive symptoms for this study was conducted using dichotomous self-survey questionnaires, the possibility of a potential information bias must also be considered. Finally, due to survey limitations, only limited occupational factors were considered.

The present study found that lower depressive symptom prevalence is associated with occupational factors like employment status and occupational type in Korean who were 55 years or older using a representative survey. The labor market has changed with the demographic trend of population aging, so employment of older people will remain an important personal, social and national issue. More public policies for an aging workforce will be needed. In the future, additional studies that address the limitations of the present study should be conducted.

## Conclusions

Using the KNHANES V, we found that the employed group showed lower late-life depressive symptom prevalence than the non-employed group among men 55 years or older. However, in older women, the risk of depressive symptoms between the employed and the non-employed group was not significantly different after adjusting for general factors. In addition, some second skill level occupations (clerks, plant and machine operators) were significantly associated with a lower risk of depressive symptoms in older men. Consequently, employment and occupation can positively support mental health in older Korean men.
